# Gel Polymer Electrolytes Based on Cross-Linked Poly(ethylene glycol) Diacrylate for Calcium-Ion Conduction

**DOI:** 10.1021/acsomega.1c02312

**Published:** 2021-06-10

**Authors:** Saeid Biria, Shreyas Pathreeker, Francielli S. Genier, Fu-Hao Chen, Hansheng Li, Cameron V. Burdin, Ian D. Hosein

**Affiliations:** Department of Biomedical and Chemical Engineering, Syracuse University, Syracuse, New York 13244, United States

## Abstract

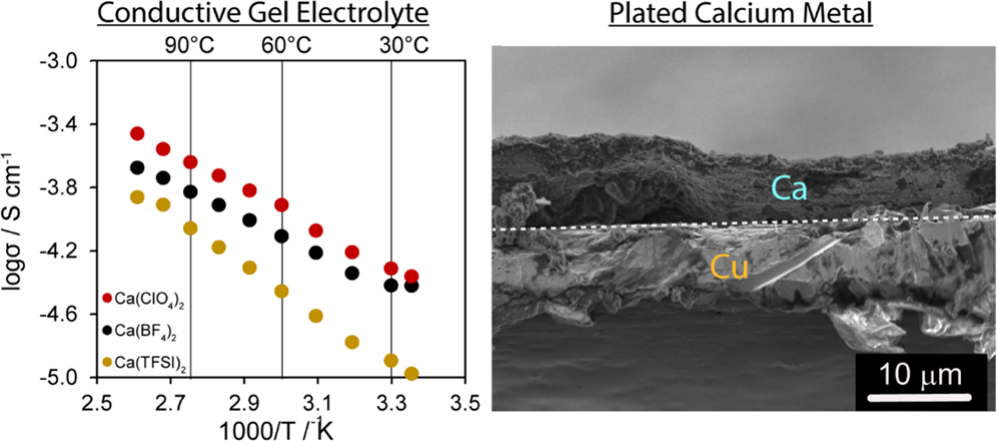

Calcium batteries are promising alternatives to lithium batteries owing to their high energy density, comparable reduction potential, and mineral abundance. However, to meet practical demands in high-performance applications, suitable electrolytes must be developed. Here, we report the synthesis and characterization of polymer gel electrolytes for calcium-ion conduction prepared by the photo-cross-linking of poly(ethylene glycol) diacrylate (PEGDA) in the presence of solutions of calcium salts in a mixture of ethylene carbonate (EC) and propylene carbonate (PC) solvents. The results show room-temperature conductivity between 10^–5^ and 10^–4^ S/cm, electrochemical stability windows of ∼3.8 V, full dissociation of the salt, and minimal coordination with the PEGDA backbone. Cycling in symmetric Ca metal cells proceeds but with increasing overpotentials, which can be attributed to interfacial impedance between the electrolyte and calcium surface, which inhibits charge transfer. Calcium may still be plated and stripped yielding high-purity deposits and no indication of significant electrolyte breakdown, indicating that high overpotentials are associated with an electrically insulating, yet ion-permeable solid electrolyte interface (SEI). This work provides a contribution to the study and understanding of polymer gel materials toward their improvement and application as electrolytes for calcium batteries.

## Introduction

Calcium batteries have been proposed as a potential post-Li alternative for electrochemical energy storage, owing to several of their attractive characteristics.^1−3^ Calcium is an earth-abundant mineral (4.1% in the Earth’s crust), with large annual production particularly in the United States, opening opportunities for low cost and domestic supply. Calcium batteries can also reach comparable gravimetric and volumetric capacities of incumbent Li-ion and emerging Li metal systems.^1^ Currently, research efforts focus on the development of stable electrode and electrolyte compositions and structures to enable reliable, long-term battery cycling. One of the challenges with calcium metal batteries is the lack of suitable electrolytes that enable stable redox activity and robust battery cycling. One primary reason for this challenge is the strong reducing (i.e., electropositive) nature of pure calcium metal, which can induce the decomposition of electrolytes into products that consequently inhibit Ca^2+^ transport, thus shutting down battery operation. Furthermore, the higher valency, larger ionic radius, and greater charge density of Ca^2+^ also result in strong coordination to the electrolyte, resulting in poor mobility, high desolvation energies at the electrode–electrolyte interface, and high overpotentials for charging. Thus far, only a few electrolytes (i.e., salt + solvent combinations) have been found effective for sustainable Ca redox activity, including Ca(BF_4_)_2_ in EC/PC, Ca(BH_4_)_2_ in THF, Ca(B(Ohfip)_4_)_2_ in DME, and Ca(BF_4_)_2_ in ionic liquids.^4−9^ Mixed cation salts have also been proposed to help provide a solid electrolyte interface permeable to Ca^2+^ or to reduce Ca^2+^ solvent coordination.^10,11^ While these works have made significant headway, there are still many opportunities for the exploration of potential electrolyte systems that may prove effective to facilitate plating and stripping of Ca for anode operation.

There is a growing interest in the development of polymer electrolytes for Ca batteries^12^ and multivalent batteries in general,^13^ in light of the aforementioned challenges with liquid electrolytes in achieving reliable Ca plating/stripping. Polymer electrolytes promise safe battery operation owing to mitigation or elimination of the flammability and volatility of the electrolyte, assistance with stabilizing metal anodes, as well as mitigation of dendritic growth,^14^ which is an issue for Ca as it is with other metals.^15^ While the lower propensity for dendritic growth and higher melting point (842 °C) make calcium melting relatively safer, nevertheless, the study and application of candidate polymer materials for their electrolyte properties for Ca^2+^ is important and can open further opportunities by demonstrating effective polymer systems.

Studies on solvent-free, solid polymer electrolytes for Ca^2+^ conduction have considered PEGDA, PEO, PTHF, and PVP–PVA.^12,16−18^ Polymer gel electrolytes, namely, a polymer host swollen with a liquid electrolyte, are also of interest owing to their higher ion mobility and greater electrolyte–electrode contact. For example, we recently showed that an ionic liquid gel electrolyte provided high ion conductivity and facilitated cycling in Ca-ion battery.^19^ Furthermore, owing to the extremely low mobility of calcium ions in a dry solid polymer host, polymer gel electrolytes are a facile approach to combine the mechanical properties of the polymer host (i.e., for separator function) and the electrolyte properties of the salt solutions toward more suitable performance. However, the number of available polymer gel candidates thus far examined for Ca batteries is lacking.

Herein, we report the preparation, properties, and electrochemical performance of a polymer gel electrolyte for Ca^2+^ conduction. The liquid component is prepared from a binary carbonate mixture of ethylene carbonate (EC) and propylene carbonate (PC) solvents with different calcium salts, calcium tetrafluoroborate (Ca(BF_4_)_2_), calcium bis(trifluoromethylsulfonyl)imide (Ca(TFSI)_2_), and calcium perchlorate Ca(ClO_4_)_2_. The gel electrolytes are synthesized by mixing poly(ethylene glycol) diacrylate (PEGDA) as the host matrix with EC/PC salt solution followed by visible light photopolymerization. The electrolytes show high ionic conductivity, stability windows >3.5 V, thermal stability, minimal Ca^2+^ coordination to the PEGDA backbone, and allow for room-temperature plating/stripping of calcium metal with promising efficiency. Plating potentials are initially commensurate with those found for liquid electrolytes; however, we observed significant increases in overpotentials associated with charge transport impedances across the solid electrolyte interface (SEI). Our work shows that plating/stripping of Ca metal is possible using a polymer gel electrolyte; however, further stabilization of the interface is needed. Hence, this work reveals challenges but also opportunities for polymer electrolytes in Ca metal batteries.

## Results and Discussion

**Figure 1:**
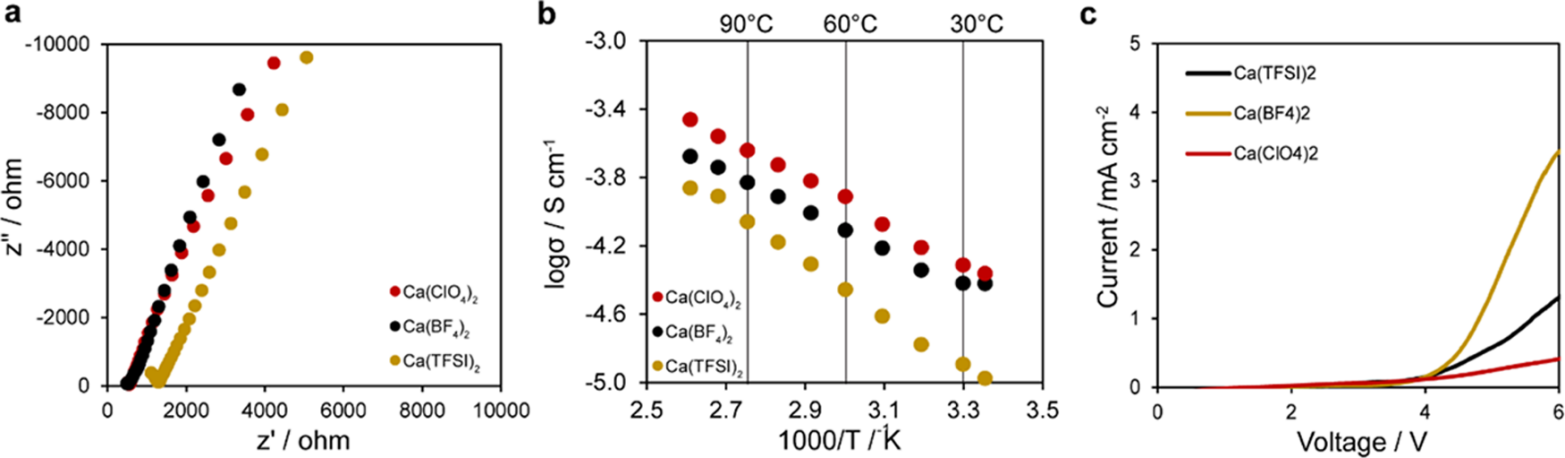
Electrochemical characterization of polymer gel electrolytes. (a) Nyquist plots of impedance at room temperature. (b) Arrhenius plots of polymer gel electrolyte conductivity. (c) Linear sweep voltammetry with Ca as both the counter and reference electrodes. Salt concentrations were 1.0 M.

Figure 1 shows the electrochemical characterization data of the polymer gel electrolytes. Ionic conductivities of each polymer gel electrolyte were determined based on impedance values obtained from the Nyquist plots shown in Figure 1a fit to an equivalent circuit model (see the Supporting Information). The small partial semicircle observed at high frequencies is associated with the bulk impedance of the electrolytes, and the following linear region at low frequencies is due to the charge-transfer resistance from the blocking effect of the stainless steel electrode and interfacial resistance between the gel electrolyte and the electrode surface. The lack of complete high-frequency semicircles in the Nyquist plots for all salts studied is most likely due to the resolution limit of the potentiostat instrument, which was unable to fully capture the response at frequencies >1 MHz. Conductivities on the order of approximately 10^–5^–10^–4^ S/cm are achieved for all salts explored (Figure 1b). Conductivities of the pure liquid electrolytes, specifically with Ca(TFSI)_2_ and Ca(BF_4_)_2_, have been measured to range from 10^–3^ to 10^–2^ S/cm (room temperature to above 100 °C),^20,21^ which places the gel electrolytes herein 2 orders of magnitude below it, yet within an acceptable conductivity range expected for gel electrolytes. The Arrhenius behavior (linear against 1/*T*) indicates that the conductivity retains its liquid-like behavior, as opposed to polymer-facilitated Ca^2+^ transport associated with polymer backbone segmental dynamics.^16,22^ The reduced conductivity, relative to pure electrolyte, could imply either some coordination of Ca^2+^ with the PEDGA host or the reduced viscosity of the gel; Raman analysis confirms that the former is not present (vide infra). Hence, reduced conductivity is most likely due to the increased viscosity induced by the cross-linked PEGDA network.

The electrolytes are stable against blocking-electrode configurations (see the Supporting Information) and provide stability windows approx. >3.5 V against calcium metal (Figure 1c). We adopted the formalism of defining the stability window based on the onset of the nonlinear portion (asymptotically tending to infinity) of the *I*–*V* curve,^7,8^ which implies a stability window of ∼3.8–4 V. Low-level currents on the order of ∼μA/cm^2^ prior to this voltage are associated with some electrolyte breakdown or side reactions, which we observed between 2 and 3.8 V, as also seen by others.^5^ The stability of ∼3.8 V is comparable to that of ionic liquid gels (with Ca(BF_4_)_2_) and liquid electrolytes consisting of alkyl fluorinated salts.^7,8,19^ This level of anodic stability is also comparable to that previously observed for EC/PC electrolytes (3.5 V)^20^ and indicates that the electrolyte may be appropriate for use in studying high-voltage cathode materials.^23−26^ It is also higher than the 3.0 V stability of Ca(BH_4_)_2_ electrolyte used to deposit Ca on a Au substrate^6^ as well as 3.0 V for Ca(BF_4_)_2_ in an ionic liquid electrolyte for a Cu substrate.^9^ The electrochemical stability characteristics observed herein are also similar to those reported for polymer electrolytes for magnesium batteries.^27,28^ Overall, the large operational window, particularly the slight increase with respect to pure liquid electrolytes, is attributed to the high oxidative stability of the cross-linked polymer, the high degree of salt dissociation, as well as the electrochemical stability of the anions.

**Figure 2:**
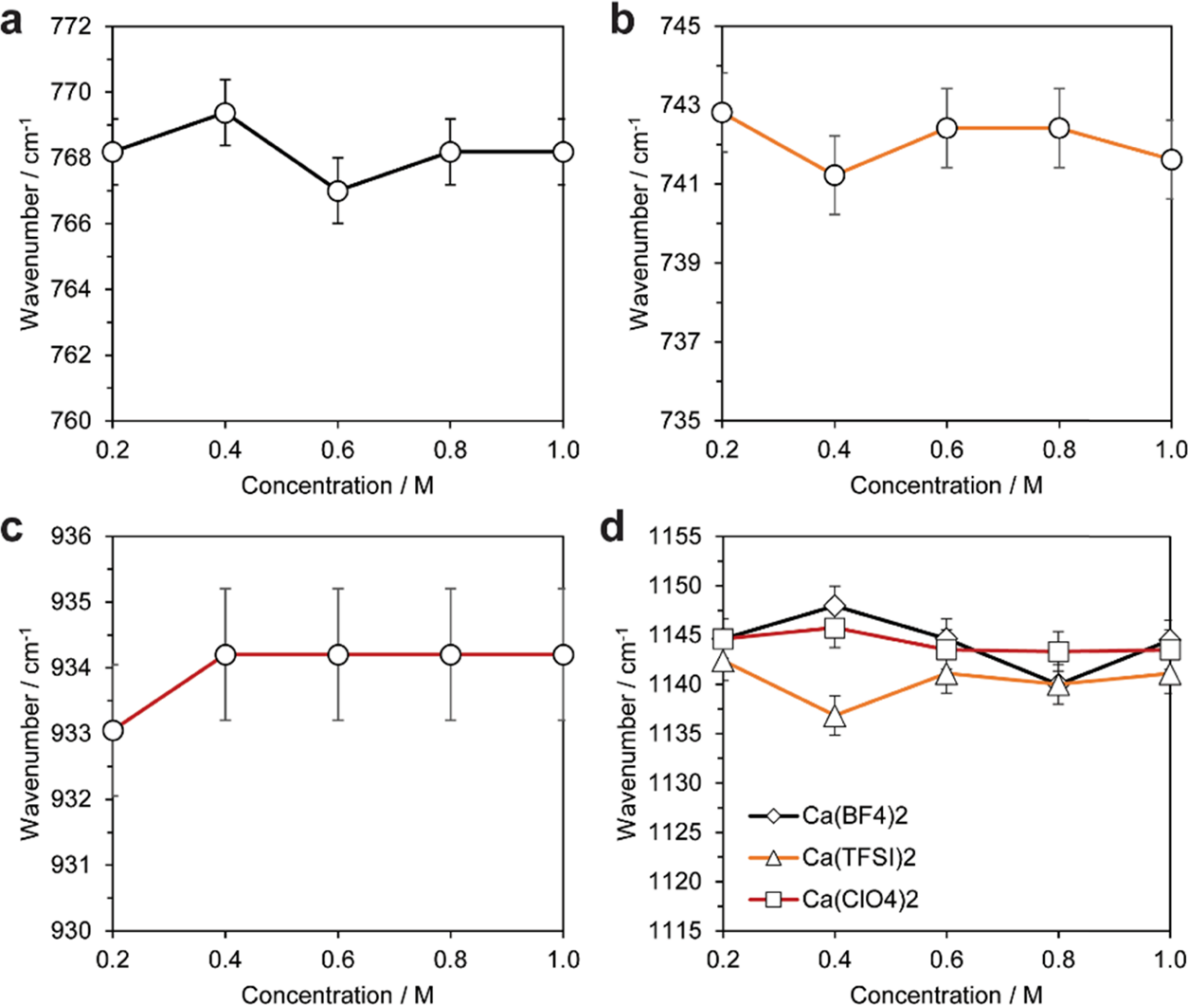
Raman spectroscopic analysis of band positions as a function of concentration in polymer gel electrolytes. (a–c) Characteristic anion band positions for BF_4_^–^, TFSI^–^, and ClO_4_^–^, respectively, in their corresponding electrolytes. (d) Characteristic band position for the vibrational mode of the C–O–C group in the PEGDA backbone.

We conducted Raman spectroscopic analysis to reveal details of the Ca^2+^ coordination environment, specifically with regard to interactions with the PEDGA host, as would be indicated by shifts in the corresponding vibrational modes of the constituent components (Figure 2). Corresponding Raman spectral data and Gaussian fits to discern Raman peak positions are provided in the Supporting Information. The salts employed herein are soluble and well coordinated with the carbonate solvents.^21^ Hence, our analysis focused on examining (1) changes to the PEGDA host as a result of coordination to Ca^2+^ and in turn (2) any effect on the solvation properties of the salts in the EC/PC solvent as revealed by the vibrational bands of the anions, both of which are assessed by examining Raman spectral band positions as a function of salt concentration. Figure 2a–c shows the Raman peak positions associated with the salt anions (BF_4_^−^, TFSI^−^, and ClO_4_^−^) for their respective electrolytes. The peak positions show no statistically significant shift with an increase in salt concentration from 0.2 to 1.0 M. The corresponding vibrational modes of the BF_4_^–^, TFSI^–^, and ClO_4_^–^ anions centered at ∼768, 742, and 934 cm^–1^, respectively, are all characteristic of free anions in the carbonate electrolyte,^29−31^ as opposed to solvent-shared ion pairs or contact ion pairs, whose peaks would be present at higher wavenumbers. Such observations are consistent with the full solubility of the calcium salts up to 1.0 M in pure solvents.^21^ We considered the well-resolved asymmetric vibrational mode of the C–O–C bond (ν(COC)_a_, ∼1142 cm^–1^) of the PEGDA host to decipher any coordination with Ca^2+^. Once again, there was no statistically significant change in the peak position when varying the salt concentration from 0.2 to 1.0 M (Figure 2d), which would indicate little if any coordination of Ca^2+^ with the PEGDA host. This is in contrast to a dry solid electrolyte with PEGDA, which shows shifts in bands associated with the C–O–C group.^16^ The lack of coordination to the ether groups of PEGDA could be owing to Ca^2+^ more strongly coordinating to the EC–PC electrolyte (particularly its C=O groups), thus reducing or eliminating any significant interactions with PEGDA. Overall, there is strong evidence that the calcium salts are present as fully soluble carbonate-based electrolytes emersed in the PEGDA host.

**Figure 3:**
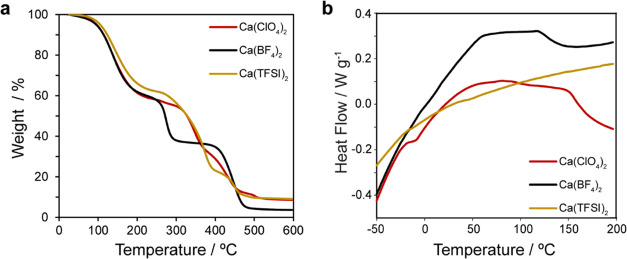
(a) TGA curves of polymer gel electrolytes using a ramp rate of 10 °C/min under a nitrogen atmosphere. (b) DSC plots of samples. Salt concentrations were 1.0 M.

We assessed the thermal stability and decomposition of the gel electrolytes with TGA and DSC (Figure 3). The initial reduction in mass up to temperatures of ∼150 °C is owing to the absorbed moisture exposure from the ambient during handling; the representative thermal response of the electrolyte is observed thereafter. Importantly, the electrolytes are stable up to temperatures of ∼200 °C, well above standard operation temperatures for batteries. At greater temperatures, mass loss is associated with thermal evaporation of the EC/PC electrolyte (between 200 and 300 °C) and followed by the thermal breakdown of the PEGDA backbone above 350 °C. The DSC curves show that the polymer electrolytes have no glass-transition temperature (Figure 3b), in accordance with the expected cross-linked, amorphous, and rubbery nature of the PEDGA host. The liquid-like, Arrhenius behavior of the electrolyte conductivity obviates any possible Vogel–Tamman–Fuelcher (VTF)-related mechanisms of conductivity associated with segmental motion,^12^ as would be expected for cross-linked materials in their rubbery state, which is a benefit of decoupling conductivity from the polymer backbone segmental motions by using a gel electrolyte. Drops in the DSC at higher temperatures are associated with solvent evaporation. TGA and DSC data were consistent among all concentrations for each respective salt explored (see the Supporting Information).

**Figure 4:**
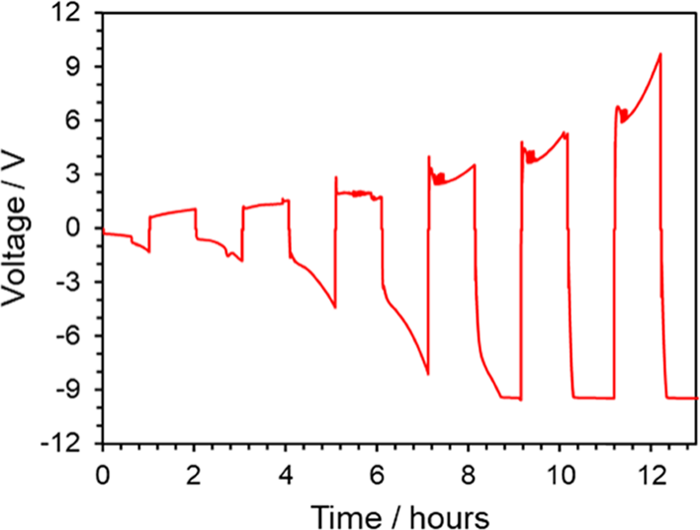
Electrochemical results for the cycling of symmetric cells (Ca//Ca) in polymer gel electrolytes with 1.0 M Ca(BF_4_)_2_. Galvanostatic cycling shown for the first 7 cycles at a current density of 2 μA/cm^2^.

To assess the capabilities of the gel polymer electrolyte to allow cycling against a Ca metal electrode, we performed galvanostatic cycling experiments in symmetric cells (Ca//Ca) using a thin (100 μm) polymer gel electrolyte. We have previously observed plating/stripping of Ca metal at room temperature using EC/PC with Ca(BF_4_)_2_;^20^ hence, we focused on exploring PEGDA with this liquid electrolyte in electrochemical experiments. Figure 4 shows galvanostatic cycling of the symmetric cell at a low-current density (2 μA/cm^2^) up to areal capacities of 1.1 mAh/cm^2^. While cycling proceeds initially within a ±1 V window in the first cycle, overpotentials to obtain the desired anodic and cathode currents continue to increase with each consecutive cycle, and eventually saturating at the maximum instrument voltage. Higher current densities showed similar results (see the Supporting Information). Lack of stable cycling at low overpotentials, which rather increase with cycling, indicates evolving processes affecting Ca^2+^ transport or redox activity.

The increasing overpotentials could be attributed to the SEI layer, charge transfer, or even electrolyte breakdown. To uncover the process, EIS measurements were first performed after each anodic and cathodic step in the GS cycling to extract impedances associated with the SEI (*R*_SEI_), charge transfer (*R*_CT_), and total cell impedance (*R*_total_) (Supporting Information). *R*_SEI_ values representing the SEI layer impedance were extracted from the *x*-intercept of the high-frequency semicircle. SEI layer impedance gradually increases with cycle number both after plating and after stripping, reaching a maximum at the 10th cycle. Some impedance values could not be extracted due to noise in the original Nyquist plots. After most cycles, SEI impedances after plating and stripping are similar in magnitude. *R*_total_ values representing total cell impedance were extracted from the *x*-intercept of the low-frequency semicircle, and *R*_CT_ representing resistance to charge transfer was calculated by subtracting *R*_SEI_ values from *R*_total_ values. Charge-transfer and total cell impedances both after plating and after stripping also increase with an increase in cycle number and appear to reach a maximum at cycle 6 for plating and cycle 7 for stripping. Notably, these maxima agree well with the gradual increase in overpotentials seen during galvanostatic cycling, suggesting that the high overpotentials required for plating and stripping are not due to high SEI layer impedance but rather due to high charge-transfer impedance and consequently high total cell impedance. *R*_SEI_, *R*_CT_, and *R*_total_ all increase with cycle number, both after plating and after stripping. However, the magnitudes of *R*_SEI_ are negligible in comparison with those of *R*_CT_ and *R*_total_. Notably, *R*_CT_ dominates the low-frequency-derived total impedance values. Furthermore, the high *R*_CT_ values may be attributed to poor electron transfer between the SEI layer and the calcium metal surface, possibly owing to slow reaction kinetics or transport limitations, thereby representing electrode polarization,^32^ which is evident from the galvanostatic curve. We speculate that *R*_CT_ increases with cycle number due to the deposition of electronically insulating products from either undetectable reduction of the PEGDA backbone or electrolyte breakdown at low potentials, thereby barring electron transfer between the electrolyte and the electrode surface. The SEI retains ion permeability, thereby allowing plating/stripping, but at very high overpotentials.

**Figure 5:**
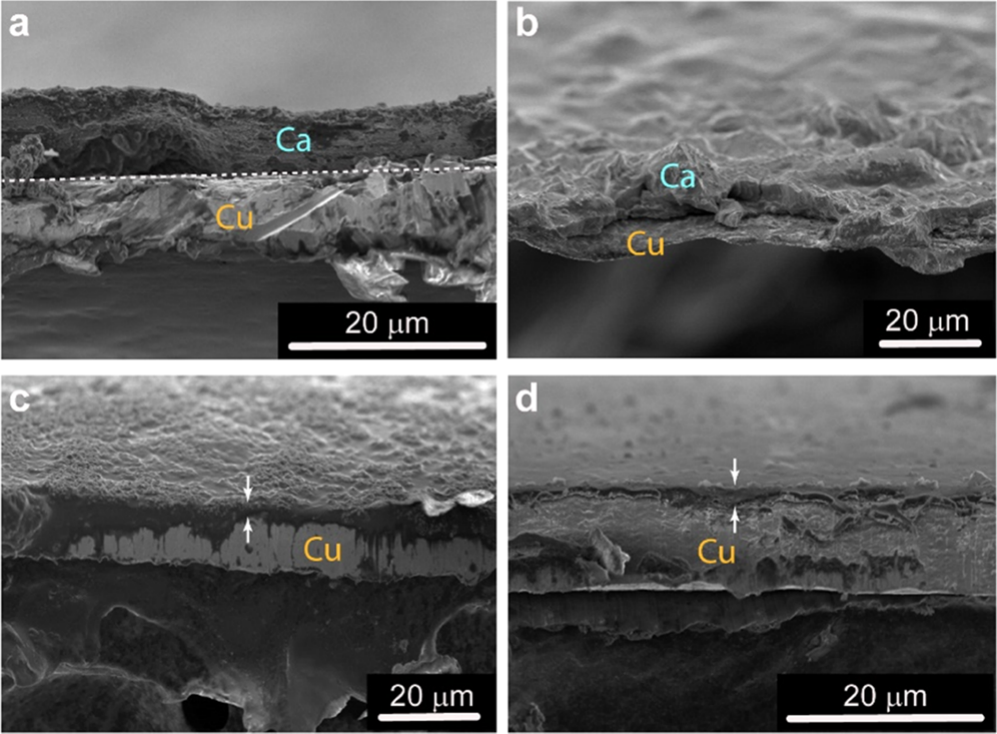
Scanning electron microscope images of the Cu working electrode. (a, b) calcium deposits after the 1st and 10th plating steps, respectively. (c, d) Cu working electrode textured by the thin SEI layer after the 1st and 10th stripping steps, respectively. Arrows indicate the cross-sectional thickness of the SEI layer. The Cu substrate is mounted on carbon tape.

To assess the plating/stripping Ca more closely, we conducted Ca plating and stripping on a Cu working electrode (Cu//Ca configuration) to examine the deposits. Plating and stripping still required significant overpotentials and were accompanied by an increase in cell impedance, as revealed by EIS spectra (see the Supporting Information). Nevertheless, Figure 5 shows SEM images of successfully plated calcium deposits present on the Cu working electrode as well as the subsequent electrochemically stripped surfaces for the 1st and 10th plating/stripping cycles. Thin ∼10 μm films of calcium are observed to be deposited over the Cu electrode. The observation of Ca deposits up to cycle 10 confirms that the SEI remains Ca^2+^-permeable, yet quite electrically insulating, thus requiring higher plating/stripping potentials. Upon stripping, the calcium deposits are removed, leaving the layer associated with the SEI produced from electrolyte breakdown. The results show that the gel electrolyte facilitates the plating and stripping of calcium. The deposits have a more continuous morphology than that observed when plating and stripping in the pure liquid EC/PC electrolyte,^4^ as well as fluorinated alkoxyborate salts in THF, but is similar to those deposited in pure THF electrolytes.^6^ Deposits using an ionic liquid electrolyte showed a smoother and more continuous deposit morphology than achieved herein.^9^ The more continuous deposits as compared to plating/stripping in the pure EC/PC liquid electrolyte are most likely owing to the gel matrix enabling more uniform transport of Ca^2+^ ions to the working electrode surface, which allows for more uniform deposition.

Currently, there are only a few reports in the literature regarding the reductive stability of PEGDA-based electrolytes. Based on the limited reports available on reversible magnesium deposition in gel polymer electrolytes, the polymers (PAN, PEO, and PVDF) appear to be stable even at low voltages,^33−35^ which reasonably suggests stability of the PEGDA backbone. Therefore, it is plausible that the electronically insulating layer is formed mainly due to decomposition of the solvent present in the electrolyte,^36,37^ which eventually causes some passivation of the Ca metal surface. All resistances are lower after stripping than after plating because the deposited layer is denser relative to the stripped layer, which can also be inferred from the SEM micrographs shown in Figure 5.

**Figure 6:**
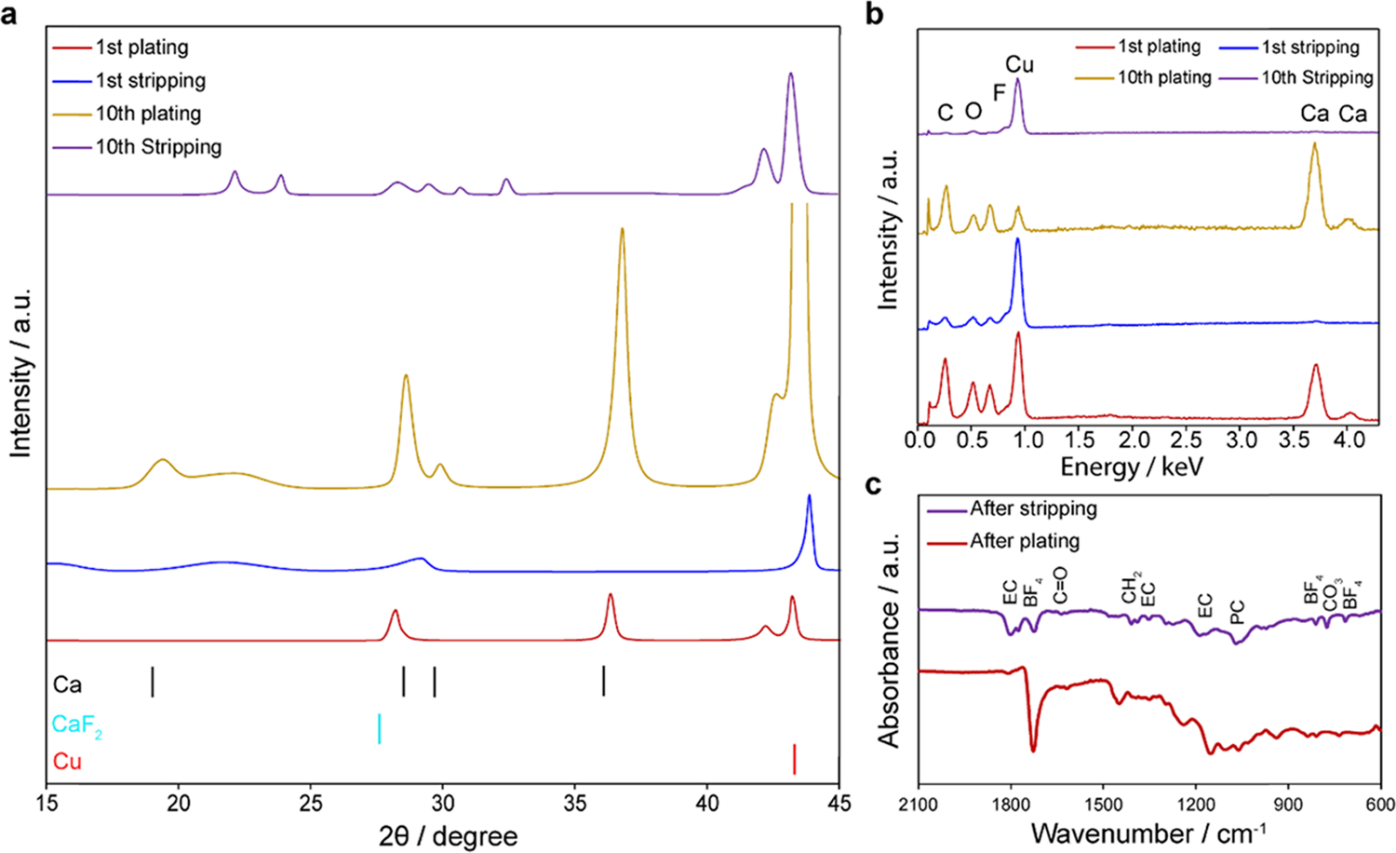
Spectroscopic analysis of plated calcium and the stripped Cu working electrode. (a) XRD and (b) EDX spectra after the 1st and 10th plating/stripping steps. (c) FTIR spectra after the 1st plating and stripping steps.

The plated deposits and stripped surfaces shown in Figure 5 were further characterized for their structure and composition. Figure 6a shows XRD spectra for the 1st and 10th plating/stripping cycles. The XRD reflections collected after the plating steps can be assigned to pure calcium metal deposits (cubic close-packed (CCP), *Fm*3̅*m*), and they attenuate after stripping (indicating Ca removal). Additional material phases are not observed. Notably, the higher XRD peak intensities in the 10th plating step as compared to the first indicate more crystal deposits. This would indicate better deposition with each plating step, possibly due to better nucleation and growth as the SEI layer stabilizes and electrolyte reduction is abated. Hence, the plating thickness did not vary significantly between plating steps, but the crystalline deposits improved. To further investigate the composition during plating and stripping, we performed EDX and FTIR analysis of the Cu working electrode after plating and stripping. EDX spectra (Figure 6b) show peaks associated with Ca after plating, which disappear upon stripping. The spectra also show that the composition of the deposits, as well as particularly the SEI (indicated from the stripped Cu surface), is composed of elements associated with the electrolyte breakdown (O, C, F, etc.). Figure 6c shows FTIR spectra of the working electrode after the 1st plating and stripping steps. The spectra are stable (i.e., similar features) between both steps, indicating a stable SEI layer in terms of composition between plating and stripping. Equivalent spectral features are found after the 10th plating and stripping steps (see the Supporting Information), indicating that this stable SEI layer is present over the course of cycling. FTIR peaks are associated with functional groups resulting from electrolyte and salt breakdown to form the SEI layer, specifically peaks associated with EC, PC, and the BF_4_^–^ anion (see the Supporting Information for peak assignments to corresponding functional groups). Once again, no peaks associated with the PEDGA matrix were found. Such decomposed electrolyte phases are found herein owing to the overpotentials beyond the stability of the electrolyte at room temperature, in contrast to other studies that employed high temperatures to enable overpotentials within the stability limit.^5^ The similar current densities achieved during the linear sweep and applied in GS cycling allow us to conclude that the low-current density observed during the former is indeed electrolyte breakdown and the SEI composition it produces should be the same as those formed during GS plating/stripping.

Overall, the results show that plating and stripping of Ca can occur using a polymer gel electrolyte. However, the necessary overpotentials are impractical. Solutions for stabilizing the Ca interface that is both electrically and ionically conductive via prepassivation or new electrolyte formulations^10^ or use of other suitable liquid electrolytes (e.g., ionic liquids, cyclic/acylic ethers, and other cyclic/acylic carbonates) may be explored to obtain reasonable plating/stripping potentials. A stable SEI should have minimal roughness, as this can otherwise lead to continuous parasitic SEI growth, which leads to low Coulombic efficiency.^38,39^ Surface images reveal significant roughness in the Ca deposits and a rough SEI layer, especially on the first plating and stripping, and relatively smoother deposits at the 10th plating step and smoother SEI layer after stripping (see the Supporting Information). It is well known that deposit and SEI morphology depend on the applied current density, electrolyte composition, salt concentration, etc.^38^ In-depth studies on the plating and stripping kinetics, electrolyte chemistry, and resultant deposits and SEI morphology are currently underway and will be reported in the future. We attribute the high overpotential specifically to the charge-transfer process, which is most likely owing to decomposition of electrolyte during the plating/stripping. Particularly during plating, an increased impedance would necessitate greater overpotential during GS cycling, forcing overpotentials beyond the stability window of the electrolyte. The precise contributing factors that lead to high impedance and large plating potentials are also the subject of current investigation. Studies on this gel electrolyte with suitable cathode materials^40^ are also underway.

## Conclusions

We have shown the synthesis and application of a promising polymer gel electrolyte consisting of EC/PC solvent and different calcium salts in a photo-crosslinked PEDGA matrix. In addition to meeting important electrolyte metrics, including high conductivity, salt dissociation, and thermal stability, Ca metal can be plated and stripped through the electrolyte; however, the high overpotentials must be addressed. This is the first demonstration of polymer gel electrolytes with carbonate solvents for calcium-ion transport and opens opportunities for further study of polymer compositions (and other polymer host chemistries) for calcium metal batteries. In-depth studies of plating/stripping, as well as investigation of prototype battery cells, are continued directions to this end.

## Experimental Section

### Materials

Poly(ethylene glycol) diacrylate (PEGDA with molecular mass, *M*_n_, of approximately 575 g/mol), photoinitiator camphorquinone (CQ), ethylene carbonate (EC), and propylene carbonate (PC) were purchased from Sigma-Aldrich. Calcium tetrafluoroborate and calcium bis(trifluoromethylsulfonyl)imide (TFSI) salts were purchased from Alfa Aesar. The initiator (4-octyloxyphenyl)-phenyliodonium hexafluoroantimonate (OPPI) was purchased from Hampford Research Inc. All of the chemicals were used as received, except for the salts that were vacuum-dried at 120 °C before use.

### Gel Electrolyte Preparation

Calcium salts with various molar concentrations were dissolved in a 1:1 by weight mixture of EC/PC as the solvent and then stirred for 24 h. Herein, reported concentrations are with respect to the solvent. Solutions of polymer electrolytes were prepared by mixing in a 1:1 ratio by weight of (1) calcium salt solutions and (2) a photocurable formulation of 96 wt % PEGDA, 2.5 wt % CQ, and 1.5 wt % OPPI, in which CQ acts as a visible light sensitizer.^41^ The mixture was stirred for 24 h while protected from light exposure in a dark room. The mixture was cured via visible light photopolymerization as thoroughly described previously.^13,16,17,19,22^

### Electrolyte Characterization

Electrochemical measurements were performed using a Solartron EnergyLab XM system. The ion conductivity of each sample was measured by AC impedance spectroscopy according to methods described previously.^16,17^ The experiments were conducted inside an argon-filled glovebox maintained at <1 ppm O_2_ and H_2_O, and the results were fitted with the instrument’s software using an equivalent circuit (see the Supporting Information). Linear sweep voltammetry between 0 and 6 V was performed using either two stainless steel plates as the blocking electrodes or a stainless steel plate as the blocking electrode and polished pure calcium metal as the nonblocking electrode. Raman spectroscopic data was obtained with a confocal microscope connected to a Raman spectrometer (Renishaw, InVia).^19,42^ Thermogravimetric analysis (TGA) and differential scanning calorimetry (DSC) studies of all samples were performed using methods described elsewhere.^16,17^

### Electrochemistry

All experiments were performed in an argon-filled glovebox maintained at <1 ppm O_2_ and H_2_O, and all measurements were performed at room temperature. A 2-electrode cell was constructed with two polished calcium disks, with the electrolyte pressed between them. Another 2-electrode cell was constructed using a copper (Cu) foil of 0.45 cm^2^ area as the working electrode and calcium metal as the counter electrode. Liquid photocurable formulation was poured into the cell and photopolymerized in situ to engulf all of the electrodes. Galvanostatic (GS) plating and stripping of calcium on the working electrode were carried out with calcium metal as both the counter and the reference electrodes, at a constant current of 1 μA (∼2 μA/cm^2^). Electrochemical impedance spectroscopy (EIS) was performed based on methods reported elsewhere.^9,19,22^

### Material Characterization

Scanning electron microscopy (SEM) was performed with a JEOL 5600 equipped with energy-dispersive X-ray (EDX) detector (accelerating voltage of 10 keV). X-ray diffraction spectra were collected with a Rigaku spectrometer using Cu Kα radiation. FTIR measurements were performed with an ATIR-FTIR spectrometer (Bruker, Alpha).

## Supporting Information Available

Conductivity plots for other salt concentrations, equivalent circuit model, electrolyte stability against blocking electrodes, Raman spectroscopic data, TGA and DSC plots for other salt concentrations, extended GS cycles for gel electrolytes, Nyquist plots, electrochemical impedances for different cycles, FTIR of working electrode, GS of plating on Cu working electrode, FTIR of deposits on Cu working electrode, SEM of deposits on Cu working electrode, and FTIR peak assignments (PDF)
