# Supplementary material for: Gel Polymer Electrolytes Based on Cross-Linked Poly(ethylene glycol) Diacrylate for Calcium-Ion Conduction
Source: ACS Omega. 2021 Jun 10;6(26):17095–102. doi: 10.1021/acsomega.1c02312 (PMC8264931; doi:10.1021/acsomega.1c02312)
Supplement: Supplementary file 1 — ao1c02312_si_001.pdf [file ao1c02312_si_001.pdf]

Gel Polymer Electrolytes Based On Crosslinked Poly(Ethylene Glycol) Diacrylate for Calcium-Ion Conduction

Saeid Biria,[1] Shreyas Pathreeker,[1] Francielli S. Genier [1], Fu-Hao Chen,[1] Hansheng Li,[1] Cameron V. Burdin,[1] Ian D. Hosein[1*]

1. Syracuse University, Department of Biomedical and Chemical Engineering, Syracuse, NY, 13244

Correspondence: idhosein@syr.edu

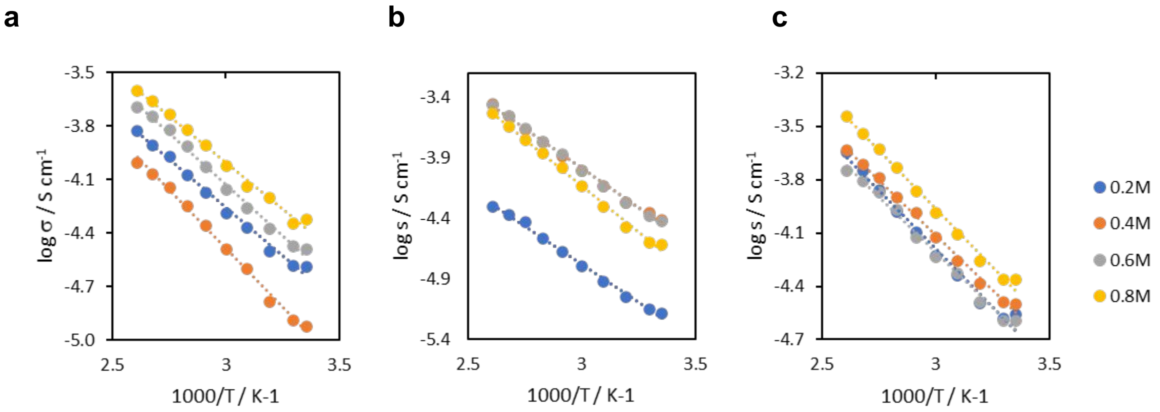

Figure S1. Arrhenius plots of the gel electrolytes containing different calcium salts. a) $Ca(ClO_4)_2$, b) $Ca(BF_4)_2$, and c) $Ca(TFSI)_2$.

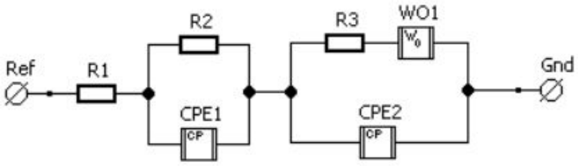

Figure S2. Equivalent circuit used to fit data acquired from electrochemical impedance spectroscopy.

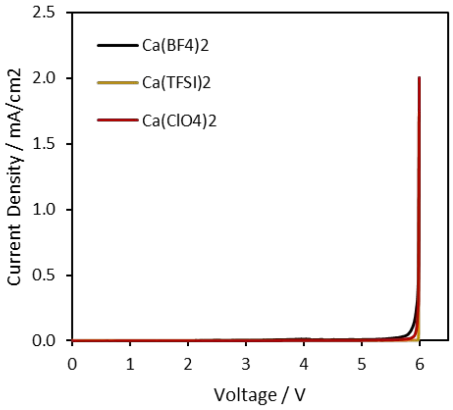

Figure S3. Electrochemical stabilities of the electrolytes, using two stainless steel blocking electrodes, achieving stability windows of ~6V.

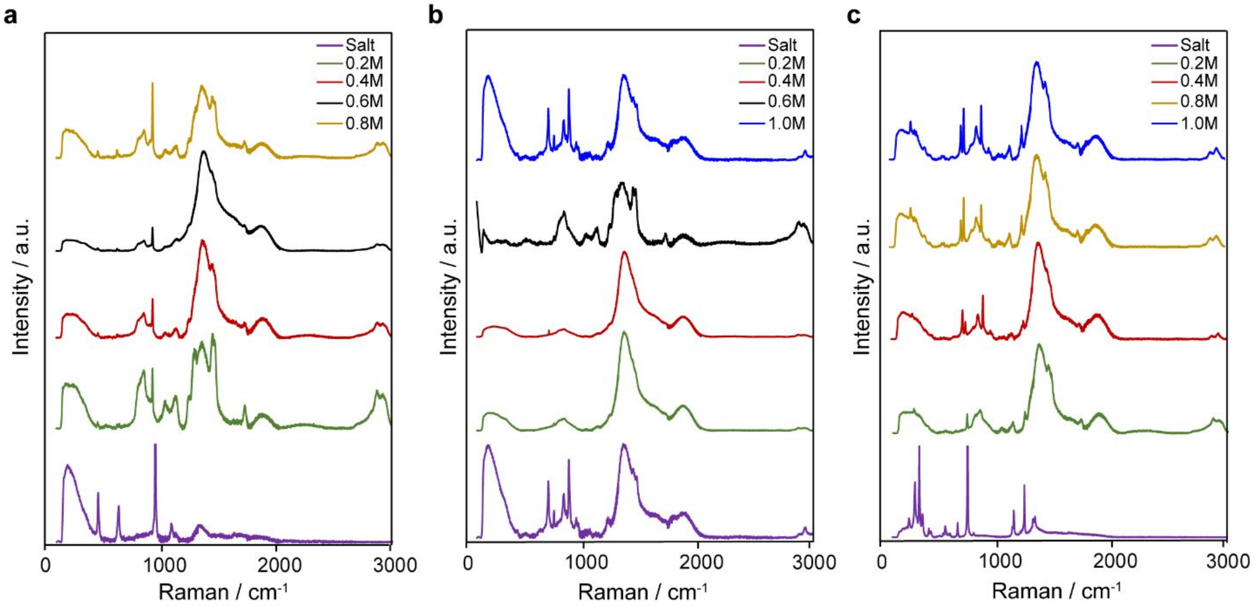

Figure S4. Raman spectra of gel electrolytes with different concentrations of calcium salts. a) $Ca(ClO_4)_2$, b) $Ca(BF_4)_2$, and c) $Ca(TFSI)_2$. Reference dried salt spectra also provided.

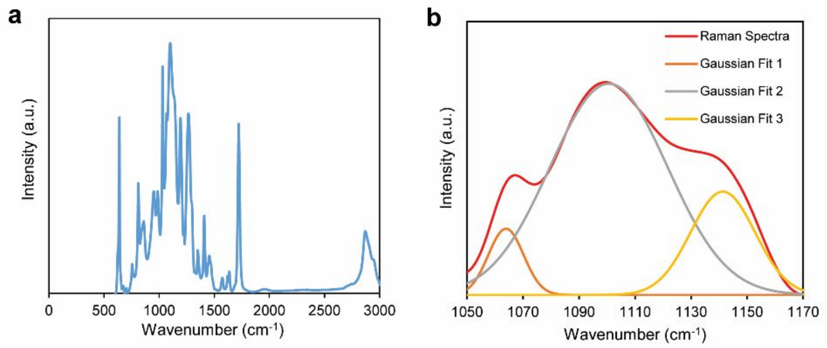

Figure S5. Raman Spectra of the pure PEGDA gel (with EC/PC and no salt) (a) full spectra, and (b) in the region showing vibrational bands resolvable with the gel electrolytes. Gaussian fits indicate the peaks positions for the $r(CH2)_a\nu(COC)_s$, $\nu(COC)_s$ and $\nu(COC)_a$ bands centered at approximately, 1060, 1101, and 1141 cm^{-1}, respectively.

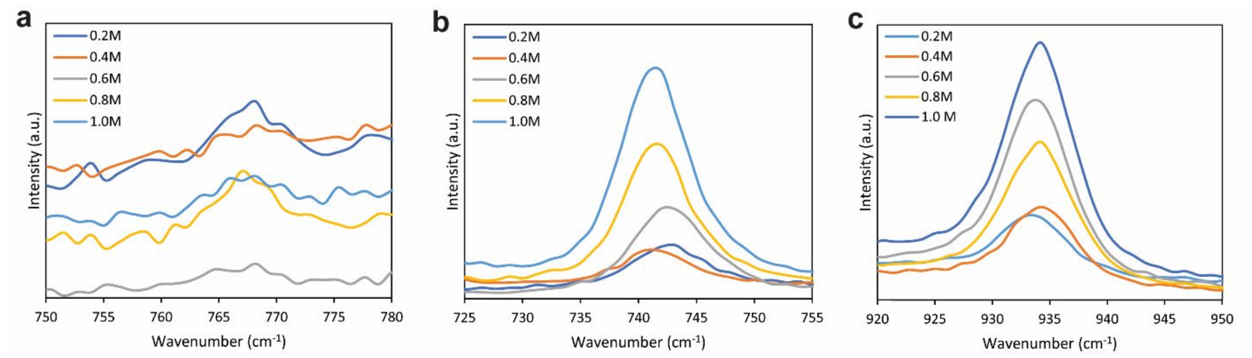

Figure S6. Raman spectra centered on the anion vibrational band for anions (a-c) BF_4^- , $TSFI^-$, and ClO_4^-, respectively, as a function of salt concentration.

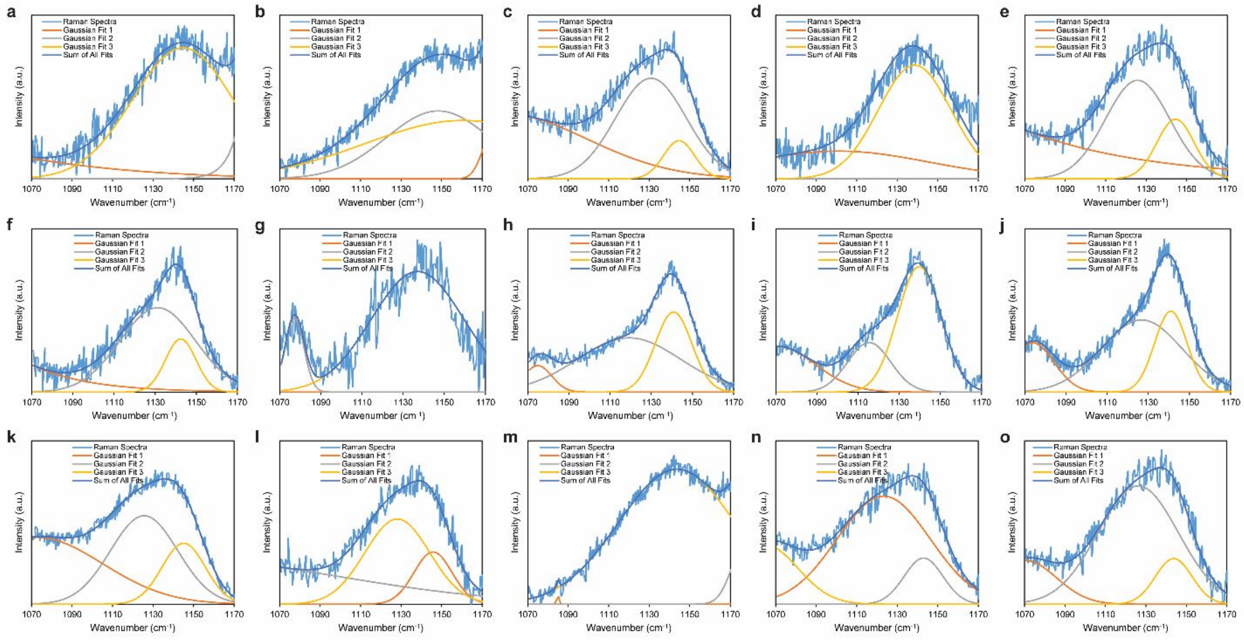

Figure S7. Raman spectra in the region of the ν(COC)a band of the PEGDA for a-e) $Ca(BF_4)_2$, f-j) $Ca(TFSI)_2$, and k-o) $Ca(ClO_4)_2$. Electrolyte concentrations are 0.2 to 1.0 for columns left to right, respectively.

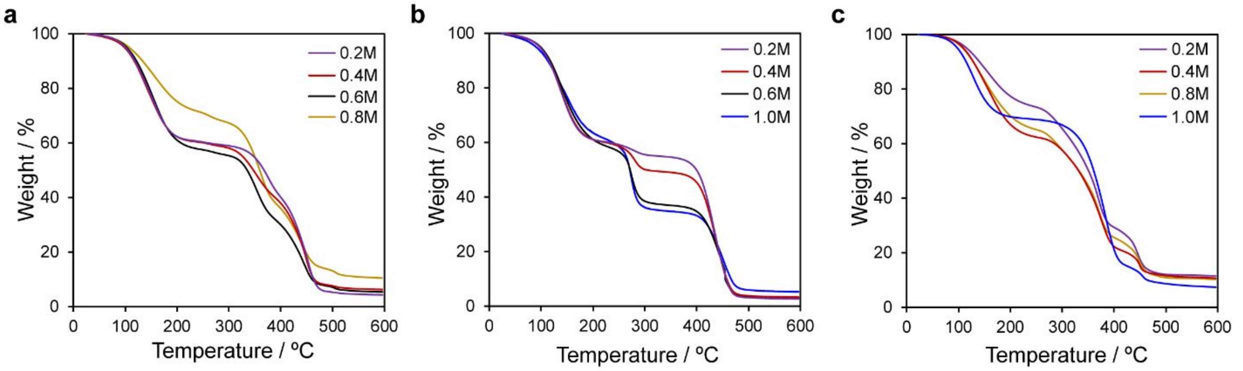

Figure S8. TGA curves of gel electrolytes with different concentrations of calcium salts. a) $Ca(ClO_4)_2$, b) $Ca(BF_4)_2$, and c) $Ca(TFSI)_2$.

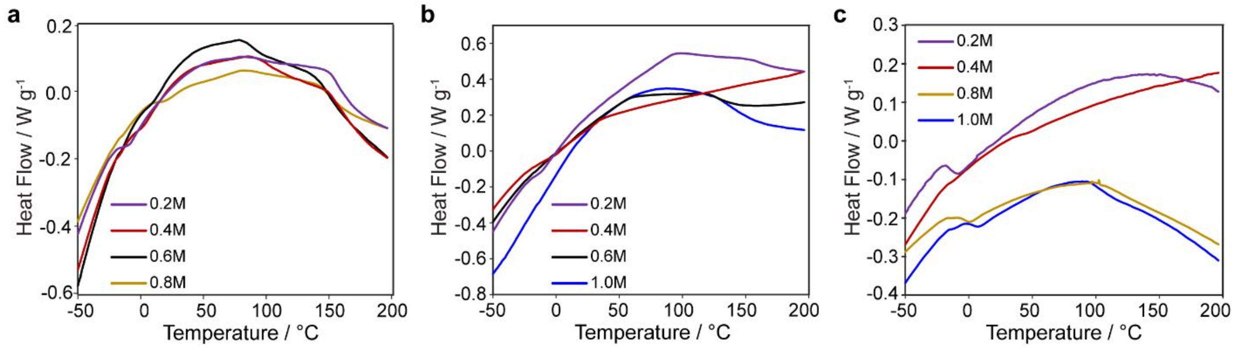

Figure S9. DSC plots of gel electrolytes with different concentrations of calcium salts. a) $Ca(ClO_4)_2$, b) $Ca(BF_4)_2$, and c) $Ca(TFSI)_2$.

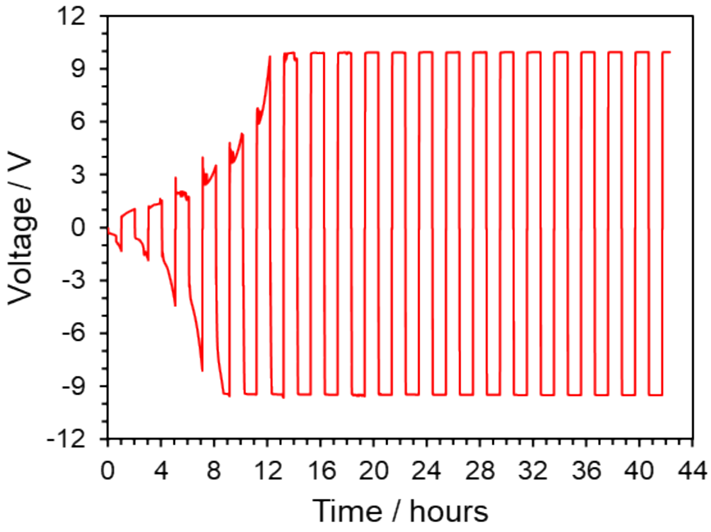

Figure S10. Galvanostatic cycling of a symmetric cell (Ca//Ca) with a PEGDA gel electrolyte with $Ca(BF_4)_2$.

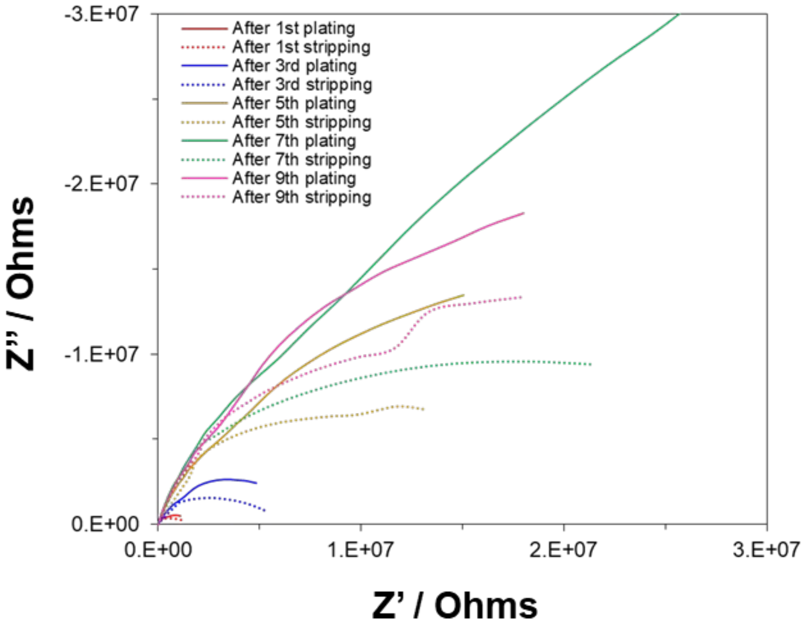

Figure S11. Nyquist plots of impedance after each plating and stripping steps with respective to the working electrode.

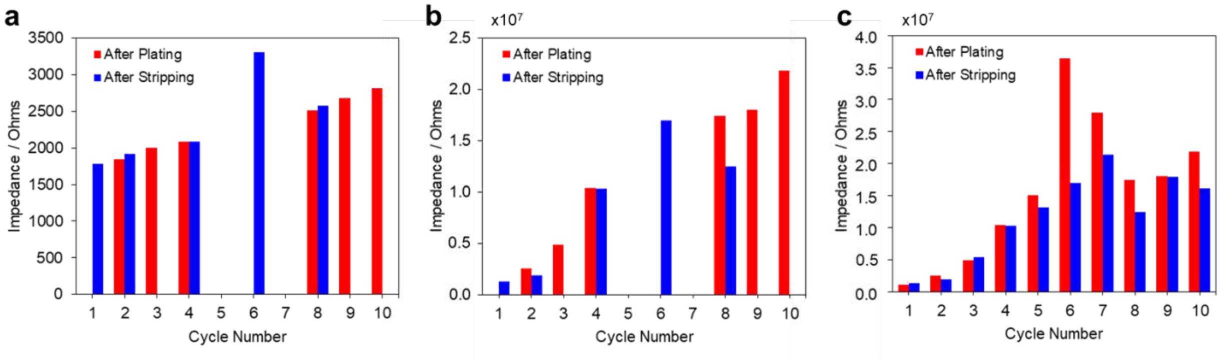

Figure S12. Impedances measured after each plating/stripping step up to 10 cycles. a) R_{SEI}, b) R_{CT}, and c) is R_{Total}. Cycles missing values is owing to the inability to fit the Nyquist plots accurately to the circuit model.

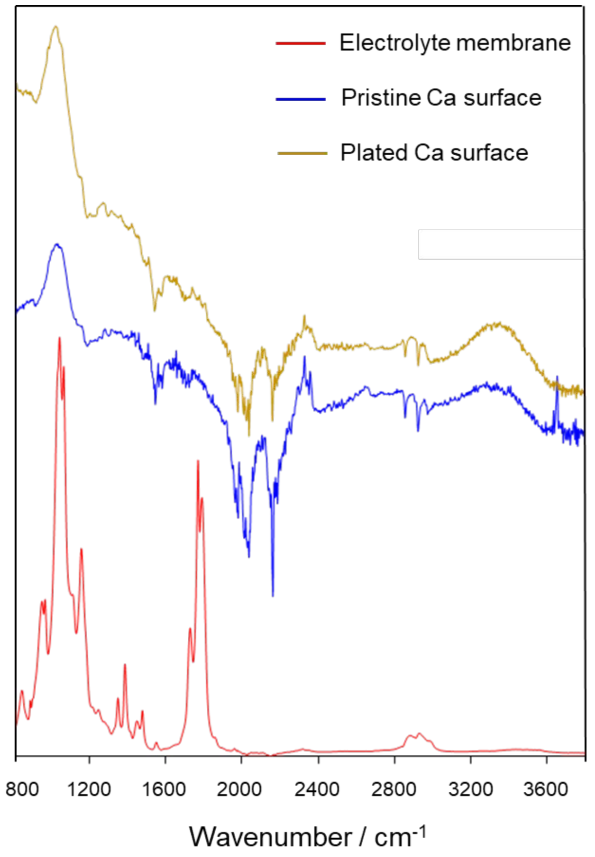

Figure S13. FTIR spectra of the PEGDA gel with $Ca(BF_4)_2$ (red), and the Ca metal surface in contact with the polymer gel electrolyte (blue) and the pristine surface (gold).

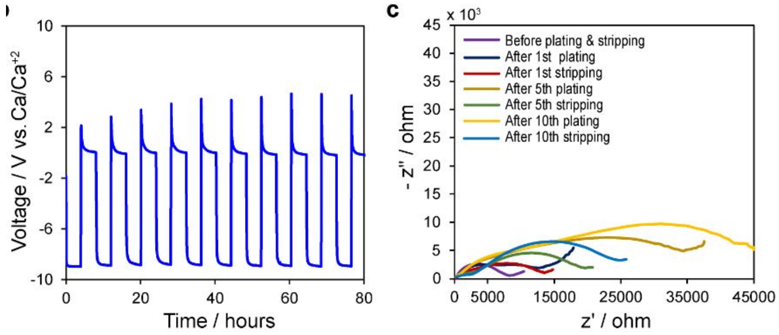

Figure S14. Electrochemical results for plating and stripping Ca metal on the Cu working electrode in gel electrolyte with 0.8 M $Ca(BF_4)_2$. (b) Plating and stripping overpotentials during galvanostatic polarization over the first 10 cycles. (c) Nyquist plots of impedance after each plating and stripping step shown in (b).

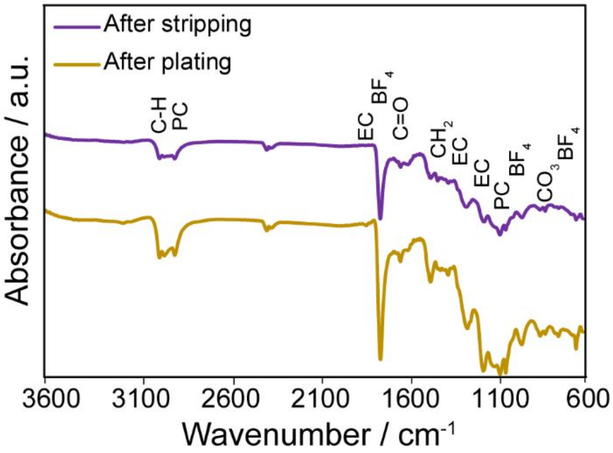

Figure S15. FTIR spectra of the Cu working electrode after the 10th plating and stripping steps in an electrolyte with 0.8 M $Ca(BF_4)_2$.

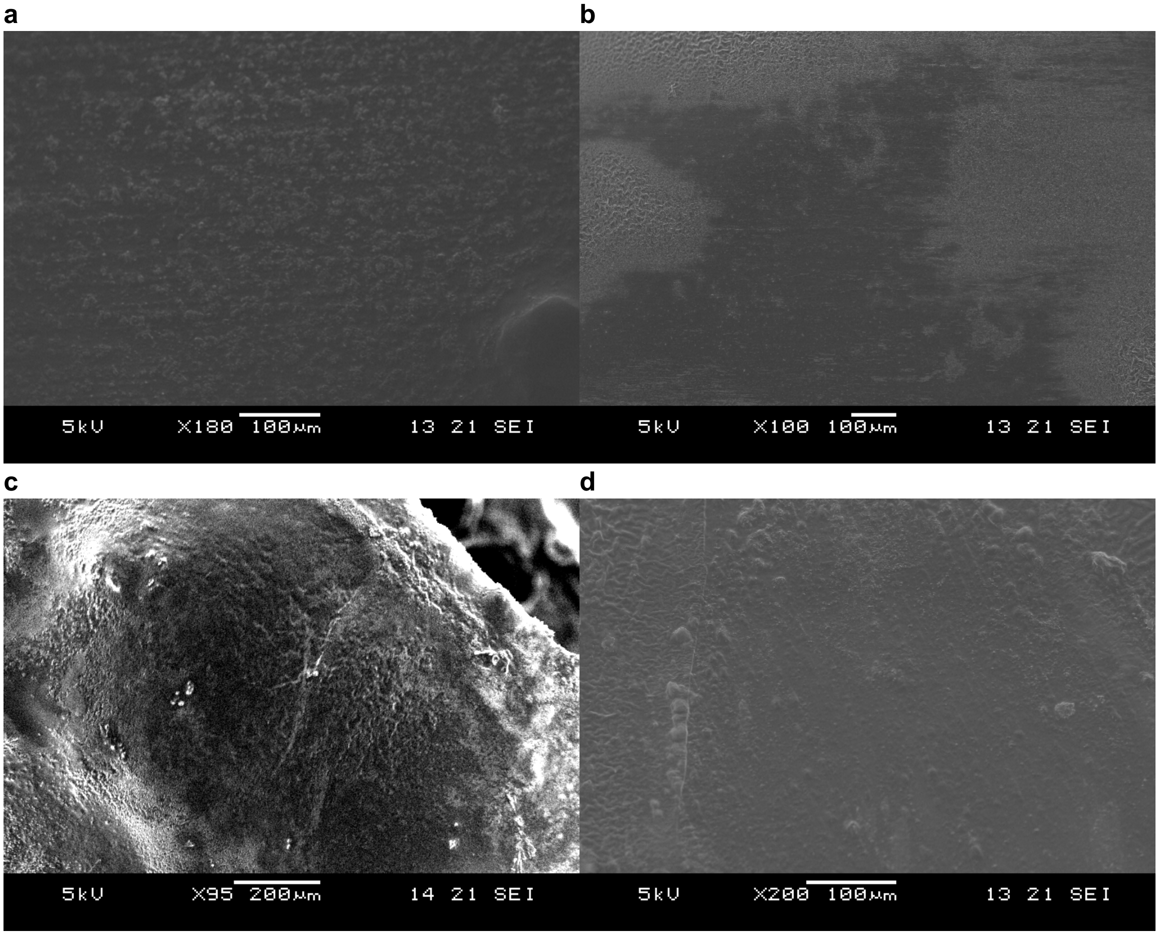

Figure S16. Electron microscopy images of the surface of the Cu working electrode. (a) 1st plating. (b) 1st stripping. (c) 10th plating. (d) 10th stripping.

Table S1. Summary of wavenumber assignment ranges corresponding to the peaks identified in FTIR analysis.

**Functional group assigned**	**FTIR (cm^{-1})**	**References**
C-H	2974	Ref. 1
PC	1041(C-O stretching)<br>2912 (C-H stretching)	NIST Chemistry WebBook, SRD 69<br>https://webbook.nist.gov/cgi/cbook.cgi?ID=C108327&Type=IR-SPEC&Index=1
EC	1124<br>1203 (CO_2 bending)<br>1791(asym. stretching)	Ref. 2<br><br>Ref. 3
C=O (asym. stretching)	1598	Ref. 3
C=O (sym. stretching)	1300	Ref. 3
CH_2 (bending)	1420	Ref. 3
CO_3 (bending)	808	Ref. 3
BF_4^-	1197, 1014	NIST Chemistry WebBook, SRD 69<br>https://webbook.nist.gov/cgi/cbook.cgi?ID=C14874705&Mask=800

REFERENCES

1. Misra, A.; Tyagi, P. K.; Singh, M. K.; Misra, D. S., FTIR studies of nitrogen doped carbon nanotubes. *Diamond Relat. Mater.* **2006,** *15*, 385-388.
2. Gatehouse, B. M.; Livingstone, S. E.; Nyholm, R. S., 636. The infrared spectra of some simple and complex carbonates. *J. Chem. Soc.* **1958**, 3137-3142.
3. Ponrouch, A.; Frontera, C.; Barde, F.; Palacin, M. R., Towards a calcium-based rechargeable battery. *Nat. Mater.* **2016,** *15*, 169-173.
